# Effect of low-intensity pulsed ultrasound on postoperative rehabilitation of rotator cuff tears: Protocol for a systematic review and meta-analysis

**DOI:** 10.1371/journal.pone.0308354

**Published:** 2024-08-15

**Authors:** Xiali Xue, Amila Kuati, Hao Fu, Qingfa Song, Qiang Liu, Guoqing Cui

**Affiliations:** 1 School of Sports Medicine and Health, Chengdu Sport University, Chengdu, China; 2 Department of Rehabilitation, Peking University Third Hospital, Beijing, China; 3 Department of Sports Medicine, Peking University Third Hospital, Beijing, China; Universiti Malaya, MALAYSIA

## Abstract

**Background:**

Rotator cuff tears are a common shoulder injury that significantly impacts patients’ daily lives and work abilities. Although surgical treatment methods for rotator cuff tears have been continuously improved with advances in medical technology, postoperative rehabilitation remains challenging. Therefore, finding effective rehabilitation treatments is crucial for improving patient prognosis and enhancing quality of life. This study will aim to systematically evaluate the impact of low-intensity pulsed ultrasound (LIPUS) on postoperative rehabilitation of rotator cuff tears, comprehensively assessing the efficacy and safety of LIPUS in postoperative recovery.

**Methods:**

This protocol will search multiple databases including PubMed/MEDLINE, Embase, Cochrane Library, CNKI, Scopus, and Web of Science to identify randomized controlled trials related to LIPUS for postoperative rehabilitation of rotator cuff tears. The search will encompass literature published from the inception of the databases up to April 2024. Methodological quality assessment and data extraction will be conducted using the Cochrane Handbook for Systematic Reviews of Interventions and PRISMA guidelines. Meta-analysis will be performed on appropriate studies using either random-effects or fixed-effects models, and subgroup analyses will be conducted to explore potential heterogeneity. Studies meeting the inclusion criteria will be included in the analysis. All analyses will be performed using Stata version 16.0.

**Results:**

The incidence of rotator cuff tear rates will be assessed by imaging techniques such as MRI or ultrasound. Pain intensity will be scored using standardized pain assessment scales, such as the Visual Analog Scale (VAS). Improved range of motion (ROM) in shoulder flexion, abduction, and rotation. Functional outcomes will be evaluated using effective measures such as Constant-Murley scores (CMS) and shoulder joint scores by American Shoulder and Elbow Surgeons (ASES). Adverse events associated with LIPUS therapy, including skin irritation, increased pain, or any other complications. Subgroup analysis will also be carried out if possible.

**Discussion and conclusion:**

Following the meta-analysis, we will assess the overall effect of LIPUS on postoperative rehabilitation of rotator cuff tears, and further explore its impact on aspects such as pain relief, functional improvement, and postoperative complications. It is anticipated that this study will provide comprehensive evidence regarding the role of LIPUS in postoperative rehabilitation of rotator cuff tears, guiding clinical practice and future research. The resultant manuscript will be submitted for publication in a peer-reviewed journal.

**Protocol registration number:**

CRD42024530798.

## Introduction

Rotator cuff tears are a common shoulder injury that often leads to pain, restricted function, and decreased quality of life [1, 2]. The incidence rate ranges from approximately 13% to 32%, increasing with age [3]. Due to the importance of the shoulder in daily activities, rotator cuff tears have a significant impact on patient’s work and social lives. With advancements in surgical techniques, particularly the widespread use of arthroscopic technology [4], surgical intervention has become one of the primary treatments for rotator cuff tears [5, 6]. Surgery aims to repair the torn tissues, providing a structural foundation for shoulder function recovery. However, the postoperative rehabilitation process still faces numerous challenges, including pain management, restoration of muscle strength, and improvement of joint mobility [7]. Therefore, in the current medical context, finding effective rehabilitation methods to promote postoperative recovery, reduce complications, and enhance patient satisfaction has become a research focus in the field of rotator cuff tears treatment [8, 9]. Effective rehabilitation methods not only accelerate the patient’s recovery process but also reduce the risk of secondary injuries and complications due to improper rehabilitation, thereby further improving the patient’s quality of life.

In recent years, Low-Intensity Pulsed Ultrasound (LIPUS) has emerged as a non-invasive physical therapy modality, showing unique advantages in promoting soft tissue injury repair and alleviating pain [10, 11]. It delivers ultrasound energy to the injured site, stimulating tissue cell proliferation and angiogenesis, thereby promoting tissue regeneration and repair [12]. Additionally, LIPUS is believed to effectively relieve pain by modulating pain conduction pathways and reducing inflammatory responses [13]. Consequently, the LIPUS application has garnered attention in the field of rehabilitation medicine.

However, despite the recognized potential of LIPUS in soft tissue injury repair and pain management, the specific effects of LIPUS in postoperative rehabilitation of rotator cuff tears remain diverse in current research findings, lacking systematic evaluation and comprehensive analysis. This study aims to comprehensively review and assess existing research evidence on LIPUS in postoperative rehabilitation of rotator cuff tears through systematic review and meta-analysis methods. By comparing results from different studies, and analyzing the effects of LIPUS on postoperative pain relief, functional recovery, and complication prevention, we aim to provide more scientifically and effectively treatment strategies for postoperative rehabilitation of rotator cuff tear patients and offer a valuable reference for clinical practice and research in related fields.

### Objectives

The primary objective of this systematic review and meta-analysis is to evaluate the effectiveness of LIPUS therapy in postoperative rehabilitation for rotator cuff tears, focusing on pain reduction, functional improvement, and the structural integrity of the repaired rotator cuff as assessed through radiological outcomes.

## Methods

### Study protocol

This study protocol adheres to the guidelines outlined in the Preferred Reporting Items for Systematic Review and Meta-Analysis Protocols (PRISMA-P) [14], as detailed in S1 Checklist. Furthermore, it has been formally registered in the PROSPERO database [15] (International Prospective Register of Systematic Reviews) under registration number CRD42024530798.

### Eligibility criteria

#### Inclusion criteria

*Population*. Studies involving adult patients (≥ 18 years) diagnosed with rotator cuff tears undergoing postoperative rehabilitation will be considered eligible.

Intervention: Studies evaluating the use of low-intensity pulsed ultrasound as a treatment modality during postoperative rehabilitation will be included.

*Comparator*. Studies with any type of comparator group (e.g., placebo, standard care, other interventions) will be eligible for inclusion.

*Outcome measures*. The primary outcome will be a composite measure including Pain Reduction: Assessed using the Visual Analog Scale (VAS). Functional Improvement: Evaluated using the Constant-Murley Score (CMS). Structural Integrity: Assessed through imaging techniques such as MRI or ultrasound. Secondary Outcomes: Range of motion (ROM) improvements in shoulder flexion, abduction, and external rotation. Functional outcomes will be evaluated through validated measures, including the American Shoulder and Elbow Surgeons (ASES) Shoulder Score. Adverse events related to LIPUS treatment, including skin irritation, pain exacerbation, or any other complications.

*Study design*. Only randomized controlled trials (RCTs) will be included.

*Language*. No language restrictions will be applied.

#### Exclusion criteria

The following types of studies will be excluded from the analysis: (1) Non-randomized controlled trials (e.g., observational studies, case series). (2) Studies not focusing on postoperative rehabilitation of rotator cuff tears. (3) Studies not evaluating the use of low-intensity pulsed ultrasound. (4) Studies without relevant outcome measures. (5) Duplicate publications or studies with insufficient data.

### Search strategy

We will conduct systematic searches across the following databases: PubMed/MEDLINE, Embase, Cochrane Library, CNKI, Scopus, and Web of Science. The search will include all literature published from the establishment of the databases up to April 2024, with no language or geographic restrictions. We will use combinations of the following keywords in our searches: ("low-intensity pulsed ultrasound" OR "LIPUS" OR "low-intensity ultrasound" OR "low-intensity pulsed ultrasonic therapy") AND ("rotator cuff tears" OR "cuff tears, rotator") AND ("postoperative rehabilitation" OR "rehabilitation" OR "physical therapy"). Taking PubMed as an example, its specific search strategy is shown in Table 1. Additionally, we will manually search citation lists of relevant journals, abstracts from key conferences, and gray literature to ensure the inclusion of all relevant studies. To ensure the comprehensiveness of the search, we will also manually search reference lists of relevant studies meeting our criteria.

**Table 1:** Search strategy for the PubMed database.

# No	Searches
**#1**	“Rotator Cuff Tear” [Mesh]
**#2**	“Rotator Cuff Tears”[Title/Abstract] OR “Cuff Tear, Rotator”[Title/Abstract] OR “Cuff Tears, Rotator”[Title/Abstract] OR “Tear, Rotator Cuff”[Title/Abstract] OR “Tears, Rotator Cuff”[Title/Abstract]
**#3**	**#**1 OR **#**2
**#4**	“Low-Intensity Pulsed Ultrasound” [Mesh]
**#5**	“Low Intensity Pulsed Ultrasound”[Title/Abstract] OR “LIPUS”[Title/Abstract] OR “Low Intensity Pulsed Ultrasound Therapy”[Title/Abstract] OR “Low-Intensity Pulsed Ultrasounds”[Title/Abstract] OR “Pulsed Ultrasound, Low-Intensity”[Title/Abstract] OR “Pulsed Ultrasounds, Low-Intensity”[Title/Abstract] OR “Ultrasound, Low-Intensity Pulsed”[Title/Abstract] OR “Ultrasounds, Low-Intensity Pulsed”[Title/Abstract]
**#6**	**#**4 OR **#**5
**#7**	“Postoperative rehabilitation”[Title/Abstract] OR "Rehabilitation"[Title/Abstract] OR "Physical therapy"[Title/Abstract]
**#8**	“Randomized Controlled Trial” [Mesh]
**#9**	“Randomized Controlled Trial” [Title/Abstract] OR “Controlled Clinical Trials, Randomized” [Title/Abstract] OR “Clinical Trials, Randomized” [Title/Abstract] OR “Trials, Randomized Clinical” [Title/Abstract] OR “Clinical trial” [Title/Abstract] OR “Clinical trials” [Title/Abstract]
**#10**	**#**8 OR **#**9
**#11**	**#**3 AND **#**6 AND **#**7 AND **#**10

### Selection process

**Fig 1:**
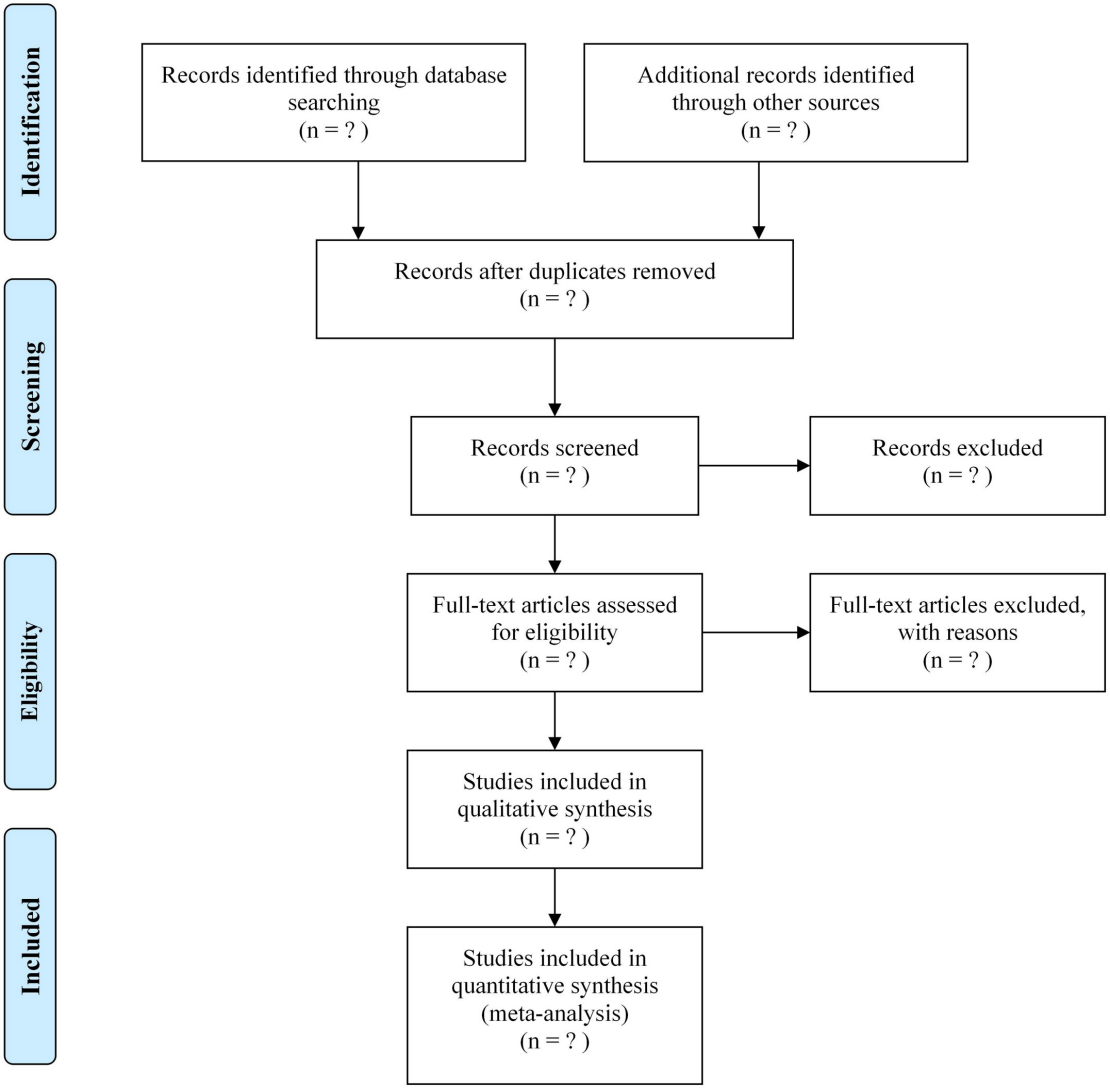
Flow diagram of the study selection process.

The literature screening will be conducted independently by two researchers to ensure the accuracy and consistency of the screening results. Initially, we will perform a preliminary screening based on titles and abstracts to exclude literature that does not meet the inclusion criteria. Subsequently, the remaining literature will undergo full-text reading, and a detailed assessment will be conducted based on the aforementioned inclusion and exclusion criteria. Any literature with disputes will be resolved through discussion or seeking the opinion of a third party. During the screening process, we will utilize the professional literature management software EndNote 21 to manage the literature and record all decisions and reasons made during the screening process [16]. This will facilitate tracking and reviewing the screening process, ensuring the transparency and reproducibility of the study. Through this rigorous literature screening process, we will ensure that the literature included in this study is of high quality and relevance, providing reliable data support for subsequent meta-analysis. The literature screening process is illustrated in Fig 1.

### Data collection process

For the literature that meets the inclusion criteria after screening, we will conduct detailed data extraction. The extracted data will include basic information about the studies (such as author(s), publication year, study design, etc.), characteristics of the study population (such as age, gender, severity of the disease, etc.), specific details of the interventions (such as ultrasound frequency, intensity, treatment duration, etc.), as well as the main outcome measures (such as postoperative recovery effects, pain scores, functional scores, etc.). We will use pre-designed tables to record this data for subsequent statistical analysis. Additionally, we will pay attention to potential biases and limitations in the literature and conduct appropriate assessment and handling. We will strive to ensure the accuracy and completeness of the data to provide a reliable basis for subsequent meta-analysis.

### Study risk of bias assessment

In this systematic review and meta-analysis, the risk of bias in the included studies will be assessed using established tools such as the Cochrane Collaboration’s Risk of Bias Tool for RCTs [17]. The assessment will be conducted independently by two reviewers, with any discrepancies resolved through discussion or consultation with a third reviewer if necessary. For RCTs, key domains including random sequence generation, allocation concealment, blinding of participants and personnel, blinding of outcome assessment, incomplete outcome data, selective reporting, and other sources of bias will be evaluated. Additionally, publication bias will be assessed using visual inspection of funnel plots and statistical tests such as Egger’s test if a sufficient number of studies are included.

### Management of missing data

In conducting this meta-analysis, we fully acknowledge the potential impact of missing data on study outcomes. Therefore, we will employ a series of strategies to manage and address these missing data. Firstly, we will make every effort to contact the original authors of the included studies to obtain any potentially missing data. This step is crucial as it directly fills in the gaps within the dataset, thereby enhancing the accuracy and reliability of the analysis. If missing data cannot be obtained from the original authors, we will consider using appropriate statistical methods for estimation or imputation. Secondly, we will employ various statistical techniques to assess and handle missing data. These methods may include multiple imputation, maximum likelihood estimation, or model-based approaches, among others. We will select the most suitable method based on the characteristics of the data and the pattern of missingness. These techniques are capable of mitigating biases and uncertainties arising from missing data to some extent. Additionally, we will conduct sensitivity analyses on the missing data to assess their impact on the overall results. This will involve comparing analysis results with and without the inclusion of missing data to determine if their absence significantly affects the conclusions. If the impact of missing data on the results is substantial, we will provide explanations in the results interpretation and discussion sections.

### Assessment of reporting biases

We will assess reporting biases using visual inspection of funnel plots and statistical tests such as Egger’s test if a sufficient number of studies (When more than 10 studies were included) are available. If asymmetry is detected, we will explore potential sources of bias and adjust our analyses accordingly.

### Subgroup analysis

We plan to stratify subgroups based on the following factors: Patient age: Younger (< 50 years) vs. older (≥ 50 years) patients. The severity of initial injury: Mild, moderate, and severe rotator cuff tears. Duration of treatment: Short-term (≤ 6 weeks) vs. long-term (> 6 weeks) treatment.

### Sensitivity analysis

We will conduct sensitivity analyses to assess the robustness of subgroup findings by altering the inclusion criteria or statistical methods.

### Grading the quality of evidence

In this meta-analysis, we will adopt the GRADE (Grading of Recommendations, Assessment, Development and Evaluation) system to conduct an objective and systematic evaluation of the quality of evidence [18]. The GRADE system integrates various factors including study design, risk of bias, inconsistency, indirectness, imprecision, and publication bias to classify the quality of evidence into four levels: high, moderate, low, and very low. We will strictly adhere to the evaluation criteria and procedures of the GRADE system, conducting detailed analysis and grading for each included study. During the assessment process, we will pay special attention to the following points: firstly, in selecting study types, we will prioritize rigorously designed randomized controlled trials with sufficient sample sizes; secondly, we will carefully assess potential risks of bias in the studies, such as selection bias and implementation bias, and adjust the quality levels of evidence accordingly; additionally, we will also focus on the consistency and precision of study results, as well as the presence of publication bias.

### Statistical analysis

All analyses will be conducted using Stata version 16.0. In the meta-analysis, effect sizes will be calculated for continuous outcomes using standardized mean differences (SMD) with 95% confidence intervals (CI), and for dichotomous outcomes using odds ratios (OR) with 95% CI. Additionally, we will employ a random-effects model for the meta-analysis to account for potential heterogeneity. Heterogeneity will be assessed using the I^2^ statistic, with I^2^ > 50% considered indicative of significant heterogeneity. In the presence of significant heterogeneity, subgroup analyses will be conducted to explore potential sources. Possible subgroup analyses will consider different age groups, gender, study quality, among other factors.

### Ethics and dissemination

The present study will use published data and does not require ethics approval.

### Dissemination of results

The resultant manuscript will be submitted for publication in a peer-reviewed journal.

## Discussion and conclusion

In recent years, LIPUS as a non-invasive physical therapy, has attracted considerable attention in the rehabilitation after rotator cuff tears [19, 20], due to its potential benefits in promoting tissue healing, relieving pain, and improving functional outcomes [21]. This protocol aims to systematically evaluate and meta-analyze the effects of LIPUS in the rehabilitation after rotator cuff tears, in order to clarify the impact of LIPUS on various outcomes, including pain levels, range of motion, strength, functional scores, and tissue healing. Given the potential advantages of LIPUS as a non-invasive treatment, this study will help clarify its role in the treatment of rotator cuff tears and provide a basis for clinical decision-making. The results of this study are expected to provide new insights into the postoperative rehabilitation of patients with rotator cuff tears. If it is found that LIPUS can significantly improve rehabilitation outcomes, this may change existing treatment guidelines and promote the clinical application of LIPUS.

While the precise mechanisms that facilitate the postoperative rehabilitation of rotator cuff tears through LIPUS remain incompletely understood, certain studies have offered potential explanations, including the enhancement of local blood circulation and acceleration of tissue repair. Further research holds the potential to delve deeper into these mechanisms, offering a firmer theoretical foundation for clinical application. For a more precise evaluation of the efficacy of LIPUS in postoperative rotator cuff tear rehabilitation, future studies are required to rigorously craft experimental protocols, expand sample sizes, and standardize assessment criteria. Moreover, the optimal timing, dosage, and duration of LIPUS treatment deserve careful consideration. Additionally, multicenter, prospective randomized controlled trials are recommended to validate current findings and further investigate the personalized therapeutic effects of LIPUS across diverse patient groups.

Furthermore, the mechanisms underlying the therapeutic effects of LIPUS warrant discussion. LIPUS is thought to exert its beneficial effects through several pathways, including stimulation of cellular activity [22], promotion of angiogenesis [23], modulation of inflammation, and enhancement of tissue regeneration [24]. By elucidating these mechanisms, we can gain insights into the physiological processes underlying LIPUS-mediated tissue repair and optimize its clinical application.

Another important consideration is the clinical relevance and practical implications of our findings. Understanding the magnitude of treatment effects and identifying potential moderators or mediators of response to LIPUS therapy can inform clinical decision-making and optimize rehabilitation protocols for patients with rotator cuff tears. Moreover, identifying gaps in the literature and areas for future research can guide the design of well-controlled trials to further investigate the efficacy of LIPUS in this context.

The study results will also examine the utilization of LIPUS in post-rotator cuff tears rehabilitation, focusing on key aspects: (1) Comparative effectiveness: Comparing LIPUS with standard rehabilitation methods like physical therapy and therapeutic exercise offers insights into its unique role and aids treatment decision-making. (2) Optimal timing and dosage: Investigating the ideal timing and dosage of LIPUS post-surgery allows tailored rehabilitation protocols, optimizing treatment outcomes. (3) Patient selection and stratification: Considering factors like tear size and patient characteristics helps identify those most likely to benefit from LIPUS, enabling personalized rehabilitation strategies. (4) Adherence: Assessing patient adherence to LIPUS therapy is crucial for interpreting findings and improving treatment outcomes through strategies like patient education and remote monitoring. (5) Safety: Ensuring the safety of LIPUS involves monitoring adverse events and considering long-term effects, contributing to a balanced risk-benefit assessment.

### Prospects

While the exact mechanisms by which LIPUS promotes postoperative rehabilitation of rotator cuff tears are not fully understood, some studies have proposed potential explanations, such as enhancing local blood circulation and accelerating tissue repair. Future research could further explore these mechanisms to provide a more solid theoretical basis for clinical application. To more accurately assess the effectiveness of LIPUS in postoperative rehabilitation of rotator cuff tears, future studies need to rigorously design experimental protocols, increase sample sizes, and standardize assessment criteria. Attention should also be paid to the optimal timing, dosage, and duration of LIPUS treatment. Additionally, multicenter, prospective randomized controlled trials should be conducted to validate our findings and further explore the personalized therapeutic effects of LIPUS in different patient populations.

### Limitations

However, this systematic review protocol has certain limitations. Variations in the brand, dosage, and intensity of LIPUS among the included studies may result in clinical heterogeneity. Differences in the timing and size of LIPUS intervention among patients also constitute potential sources of clinical heterogeneity. Discrepancies in treatment protocols and outcome assessment methods may also contribute to heterogeneity.

## Conclusions

In conclusion, this systematic review and meta-analysis protocol will provide a comprehensive synthesis of the current evidence regarding the effectiveness of LIPUS in postoperative rehabilitation following rotator cuff tears. By synthesizing data from existing studies, we aim to elucidate the therapeutic potential of LIPUS and its impact on various clinical outcomes. Our findings will inform clinical practice, guide rehabilitation protocols, and identify avenues for future research aimed at optimizing the management of rotator cuff tears. Ultimately, this review has the potential to improve patient outcomes and enhance the quality of care for individuals undergoing rotator cuff tears repair.

## Supporting information

S1 Checklist PRISMA-P (Preferred Reporting Items for Systematic Review and Meta-Analysis Protocols) 2015 checklist: Recommended items to address in a systematic review protocol. (DOC)
